# Localization of nitric oxide–producing hemocytes in *Aedes* and *Culex* mosquitoes infected with bacteria

**DOI:** 10.1007/s00441-024-03862-1

**Published:** 2024-01-19

**Authors:** Stella Bergmann, Emily Graf, Pascal Hoffmann, Stefanie C. Becker, Michael Stern

**Affiliations:** 1 Institute for Physiology and Cell Biology, University of Veterinary Medicine Hannover, 30173 Hannover, Germany; 2 Institute for Parasitology, University of Veterinary Medicine Hannover, 30559 Hannover, Germany

**Keywords:** Nitric oxide, Hemocyte, Immunity, Nervous system, Infection, Insect, Mosquito

## Abstract

Mosquitoes are significant vectors of various pathogens. Unlike vertebrates, insects rely solely on innate immunity. Hemocytes play a crucial role in the cellular part of the innate immune system. The gaseous radical nitric oxide (NO) produced by hemocytes acts against pathogens and also functions as a versatile transmitter in both the immune and nervous systems, utilizing cyclic guanosine monophosphate (cGMP) as a second messenger. This study conducted a parallel comparison of NO synthase (NOS) expression and NO production in hemocytes during *Escherichia coli* K12 infection in four vector species: *Aedes aegypti*, *Aedes albopictus*, *Culex pipiens molestus*, and *Culex pipiens quinquefasciatus*. Increased NOS expression by NADPH diaphorase (NADPHd) staining and NO production by immunofluorescence against the by-product L-citrulline were observed in infected mosquito hemocytes distributed throughout the abdomens. NADPHd activity and citrulline labeling were particularly found in periostial hemocytes near the heart, but also on the ventral nerve chord (VNC). Pericardial cells of *Ae. aegypti* and *Cx. p. molestus* showed increased citrulline immunofluorescence, suggesting their involvement in the immune response. Oenocytes displayed strong NADPHd and citrulline labeling independent of infection status. This comparative study, consistent with findings in other species, suggests a widespread phenomenon of NO’s role in hemocyte responses during *E. coli* infection. Found differences within and between genera highlight the importance of species-specific investigations.

**Supplementary Information:**

The online version contains supplementary material available at 10.1007/s00441-024-03862-1.

## Introduction

As climate change leads to shifts in the geographic range of insect species, the importance of vector control efforts and an understanding of vector competence factors becomes more pressing (Tabachnick 2010; Mojahed et al. 2022). While there is ample literature about *Anopheles* mosquitoes and malaria and the relevance of nitric oxide (NO) in this context (Hillyer and Estévez-Lao 2010; Luckhart et al. 1998; Peterson et al. 2007; Herrera-Ortiz et al. 2011; Jones et al. 1996), only sparse information on mosquito species relevant in Europe is available. Invasive species like *Aedes albopictus* and *Aedes aegypti* increase the risk of dengue, chikungunya, and Zika virus spreading in Europe (Sabatini et al. 1990; Seixas et al. 2013; Akiner et al. 2016; Beltrame et al. 2007). Additionally, European *Culex pipiens* have been shown to be able to transmit West Nile virus or Rift Valley fever virus (Engler et al. 2013; Vloet et al. 2017; Fros et al. 2015). Therefore, more insights about their immune system and influences on their vector competence are needed.

Insects have an open circulatory system, which allows the hemolymph to spread potential pathogens throughout the body cavity (League and Hillyer 2016; King and Hillyer 2013; Yan and Hillyer 2020). Mosquitoes, like all insects, lack an adaptive immune system and rely on innate immunity, which can detect conserved motifs like bacterial cell wall components (Hillyer 2016; Schmidt et al. 2001). The immune system has cellular and humoral components (Nappi et al. 2000; Carton and Nappi 1997). Hemocytes drive the cellular response, circulating or attaching to tissues, with sessile hemocytes found on the abdominal wall, tracheae, and near the heart in mosquitoes forming distinct patterns especially in the periosteal regions (King and Hillyer 2013; Yan and Hillyer 2020) or near the ventral nerve chord as previously shown in locusts (Bergmann et al. 2021). Hemocytes can phagocytose foreign particles or encapsulate them, often accompanied by a melanization reaction (Strand and Pech 1995; Zhao et al. 1995). Also, pericardial cells and oenocytes were shown to have implications for the immune response of insects (Hernández-Martínez et al. 2013; Cardoso-Jaime et al. 2021; Huang et al. 2019; Gomes et al. 2021).

One important factor for the successful elimination of pathogens in both insect and vertebrate immune systems is the gaseous radical NO. In *Anopheles* mosquitoes as well as vertebrate hosts, NO contributes to the control of bacterial and malaria infection (Hillyer and Estévez-Lao 2010; Luckhart et al. 1998; Peterson et al. 2007; Herrera-Ortiz et al. 2011; Jones et al. 1996). NO, as part of the humoral immune response, is synthesized in hemocytes and fat body from L-arginine by the NO synthase (NOS) with NADPH as a cofactor, producing equimolar amounts of L-citrulline as a by-product (Nappi et al. 2000; Rivero 2006; Martinelli et al. 2002). The gaseous properties of NO allow it to readily diffuse through membranes, making it an efficient signaling molecule serving multiple functions beyond the immune system as a neurotransmitter with cyclic guanosine monophosphate (cGMP) as a second messenger (Rivero 2006; Bicker 1998). The NO/cGMP pathway is involved in central information processing in the olfactory and visual systems, modulation of vesicle release at the neuromuscular junction, and learning and memory formation (Elphick et al. 1995, 1996; Bicker 2001; Kuntz et al. 2017; Cayre et al. 1994).

The presence of NO as a messenger both in the nervous system and the immune system during infections raises the possibility of its influence on perception and behavior (Bergmann et al. 2023). Altered behaviors contributing to vector competence were found in mosquitoes during arboviral and bacterial infections (Lima-Camara et al. 2011; Tallon et al. 2020; Bennett et al. 2008; Lee et al. 2000; Cator et al. 2013).

Understanding the immune-nervous system crosstalk is of significant interest, particularly the potential role of NO as a mediator. We examined four different vector species that are relevant for the transmission of arboviruses in Europe using NADPH diaphorase (NADPHd) staining to investigate the location of NOS by fixation-resistant reductase domain activity (Ott and Elphick 2003; Bredt et al. 1991) and anti-citrulline immunofluorescence to detect NO production in mosquito hemocytes. Comparing four vector species revealed shared patterns in hemocyte reactions to *E. coli* infection. We observed that an immune reaction can extend to the ventral side of the abdomen, with NOS and NO-producing hemocytes even attaching to the nervous system. Compared to *Ae. albopictus* and *Cx. p. quinquefasciatus*, stronger immune responses were observed in *Ae. aegypti* and *Cx. p. molestus* regarding NOS-containing and NO-producing hemocytes. Moreover, their pericardial cells show infection-induced NO production suggesting an involvement in immune response. On the other hand, oenocytes expressing strong NADPHd and anti-citrulline labeling in all investigated species appear not to be infection-dependent. Our findings highlight the need for species-specific investigations in understanding vector immunity for effective disease prevention, particularly given differences observed within the same genus.

## Material and methods

### Mosquito rearing and infection

Mosquitoes were reared in the Institute of Parasitology (University of Veterinary Medicine Hannover). In the following experiments, females of four different species were used about 1 to 2 weeks after final molt: *Culex pipiens* biotype *molestus*, *Culex pipiens* biotype *quinquefasciatus*, *Aedes albopictus*, and *Aedes aegypti*. The colonies were maintained as already reported in Heinig-Hartberger et al. (2023). In brief, the *Culex* colonies were maintained at 26 °C and the *Aedes* colonies at 28 °C both at a relative humidity of 45–75% and with a photoperiod of 16:8 (light/dark) with a 1-h twilight period at dusk and dawn. Adult mosquitoes were fed ad libitum with an 8% fructose solution and dog blood once a week.

For infection, an overnight culture of *Escherichia coli* K12 in Luria-Bertani’s broth medium was prepared at 37 °C and colony-forming units (CFU) were determined by plating serial dilutions and counting colonies after 1 day. Mosquitoes were infected with a median dose of 3.7 × 10^5^ CFU of live *E. coli* (doses ranging between 2.16 and 16.6 × 10^5^ CFU) by injecting the overnight culture with a volume of 209.7 nl into the thorax using fine glass capillaries and the Nanoject II automatic nanoliter injector (Drummond, Broomall, PA, USA).

During experiments, mosquitoes were maintained at ambient conditions in *Drosophila* tubes 150 ml (Greiner Bio-One GmbH, Frickenhausen, Germany) with a sucrose solution-soaked *Drosophila* plug ⌀ 50 × 30 mm (Greiner Bio-One GmbH, Frickenhausen, Germany) at the bottom of the tube to provide food and moisture at the same time.

If not stated otherwise, all chemicals were bought from Merck (Darmstadt, Germany).

### Dissection

After a 24-h incubation period, mosquitoes were briefly anesthetized with CO_2_ and placed on ice. Abdomens were cut off close to the thorax with small scissors. Further dissection was done similar to Stern and Bicker (2008) on microscopy slides with basins of hardened SYLGARD (SYLGARD 184 Silicone Elastomer Kit, Dow Europe GmbH, Wiesbaden, Germany) (inner proportions of basin approx. 0.5 × 0.5 cm). The basins’ ground was covered with a layer of liquid SYLGARD base and filled with cold phosphate-buffered saline (PBS, pH 7.4). The abdomens were stuck on the liquid SYLGARD base and cut along the pleural membrane on one side to spread them out on the SYLGARD layer, since mosquitoes are delicate insects and fillet preparations succeed best without applying minutiae. The gut, ovaries, and Malpighian tubes were removed. After dissection, the abdomens were immediately fixated.

### NADPH diaphorase activity staining

For the diaphorase staining, abdomens were fixated in −18 °C cold methanol/formaldehyde 38% 9:1 after Ott and Elphick (2002) for 3 min on ice and subsequently kept in cold PBS on ice. Alternatively, abdomens were fixated with paraformaldehyde 4% (PFA) for 5 min. Abdomens were washed with cold Tris buffer 0.1 M with 0.1% Triton X-100 (Tris-T, pH 8.0) for 5 min and afterwards stained with the staining solution (2 mg β-nicotinamide adenine dinucleotide 2′-phosphate (NADPH) and 2 mg nitrotetrazolium blue in 10 ml Tris-T) at room temperature in a dark place for approx. 60 min. Then, preparations were washed three times with PBS for 5 min and subsequently with *aqua dest*. to remove most of the remaining Tris buffer, which can induce crystal formation in cedar wood oil. Abdomens were further washed with methanol/acetic acid 3:1 for 1 min and afterwards three times with methanol for 1 min. Preparations were covered with cedar wood oil, and methanol was allowed to evaporate for approx. 15 min before the SYLGARD basins were removed and the slides were covered with coverslips. Abdomens fixated with PFA were not washed with methanol and instead cleared with 50% glycerol for 30 min and coverslipped in 90% glycerol.

### Anti-citrulline immunofluorescence

For the immunofluorescence labeling, abdomens were fixated with PFA with 0.05% glutaraldehyde for 10 min and subsequently permeabilized with PBS with 0.1% Triton X-100 (PBS-T) and 0.3% saponin for 30 min. Preparations were then washed with PBS-T for 10 min and blocked with PBS-T with 5% normal goat serum (BIOZOL Diagnostica Vertrieb GmbH, Eching, Germany). The first antibody mouse-anti-citrulline (Martinelli et al. 2002)—a kind gift by G.A. Holstein and G.P. Martinelli, New York, RRID: AB_2314197 – 1:200 in blocking solution—was applied overnight at 4 °C. Abdomens were washed twice with PBS-T for 5 min, and afterwards, secondary antibody goat-anti-mouse Alexa Flour 568 (Abcam, Cambridge, UK) 1:333 together with streptavidin Alexa Fluor 488 (Invitrogen, Darmstadt, Germany) 1:200 and 4,6-diamidino-2-phenylindole dihydrochloride (DAPI) 1:500 was applied in blocking solution overnight at 4 °C. Then, abdomens were washed with PBS-T for 5 min and rinsed with *aqua dest*. Preparations were cleared with 50% glycerol for 30 min and coverslipped in 90% glycerol.

### Incubation with L-citrulline

To investigate the uptake of the amino acid L-citrulline into the pericardial cells, anesthetized female *Cx. p. molestus* mosquitoes were dissected in locust Ringer (NaCl 150 mM, KCl 3.1 mM, MgCl_2_ 1 mM, CaCl_2_ 5.4 mM, NaOH 2 mM, TES 5 mM, glucose 5 mM, sucrose 100 mM, pH 7.2) at room temperature. Abdomens were dissected as stated before in SYLGARD basins. Naive and *E. coli* (2 × 10^6^ CFU, heat-inactivated for 5 min at 95 °C) injected mosquitoes were used as controls. The abdomens were incubated with locust Ringer (control), or with 50 µM, 5 µM, and 500 nM L-citrulline added for 1 h. Subsequently, abdomens were fixated and labeled with the anti-citrulline antibody as stated before using goat-anti-mouse Alexa Flour 488 (Invitrogen, Darmstadt, Germany) as secondary antibody.

### Image acquisition and evaluation

Images were acquired with an Axiocam 506 color camera linked to fluorescence microscope Axioskop and ZEN 2012 blue edition software (Carl Zeiss Microscopy, Oberkochen, Germany). Images of abdomens were checked for NADPHd- and citrulline-positive hemocytes and pericardial cells as well as for brown, melanized inclusions. Abdominal halves were defined as positive when at least three L-citrulline immunofluorescence-positive or NADPHd-positive hemocytes were present. Abdominal halves were regarded as melanized when at least ten small melanized spots (under approx. 150 µm) or one large melanized spot (above approx. 150 µm) were present. Images of pericardial cells and oenocytes were taken at × 20 magnification, equal excitation intensity, and exposure settings to acquire comparable images for evaluating anti-citrulline immunofluorescence intensity in ImageJ 1.51q (Rasband, W.S., ImageJ, US National Institutes of Health, Bethesda, MD, USA). Images of pericardial cells were randomized and evaluated by a second, blinded person. Of every analyzed individual, one image section of the dorsal and the ventral abdomen were taken. Cells must not be covered by objects. Three cells of every image were measured by marking the region of interest with the circle tool covering most of the cell and determining the mean gray value. Intensity variability of pericardial cells in *E. coli*-infected mosquitoes compared to naive was calculated from the range of *E. coli* values divided by range of naive values. Elevated intensity of pericardial cells of *E. coli*-infected mosquitoes compared to naive was calculated as the fraction of intensity values from infected mosquitoes above the 75% percentile of values of naive mosquitoes.

## Results

**Fig. 1:**
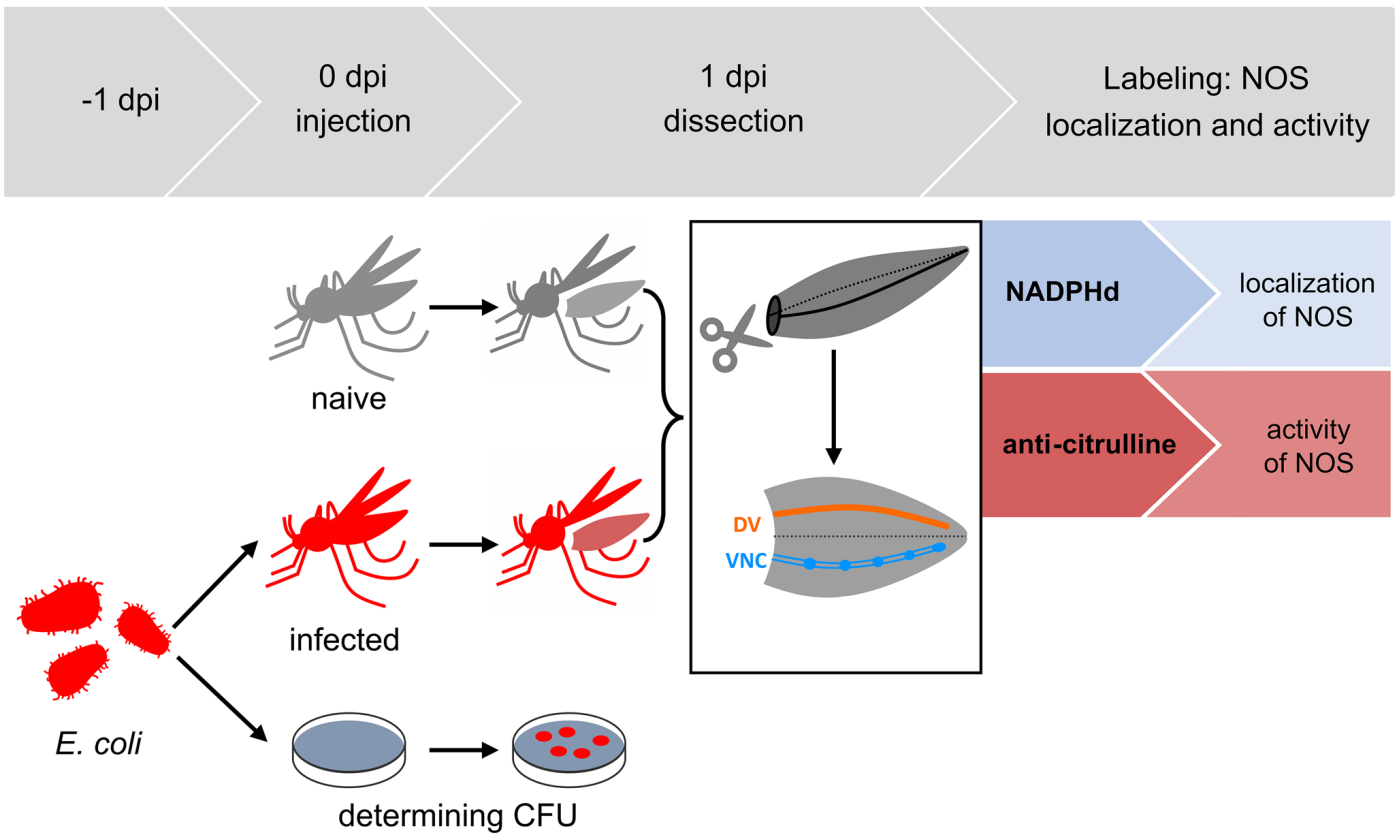
Experimental schedule. A median dose of 3.7 × 10^5^ CFU of live *Escherichia coli* (*E. coli*) of an overnight culture was injected in *Culex pipiens molestus*, *Culex pipiens quinquefasciatus*, *Aedes albopictus*, and *Aedes aegypti* mosquitoes. Control mosquitoes were left naive. Parallel serial dilutions were done to determine the colony-forming units (CFU) of the bacterial culture the next day. At 1 day post infection (dpi), anesthetized naive and infected mosquitoes were dissected. Abdomens were cut off and opened by cutting along the pleural membrane to expose the ventral nerve chord (VNC) and the dorsal vessel (DV). To investigate the location and the activity of the nitric oxide synthase (NOS) in the hemocytes and pericardial cells, NADPHd staining and anti-citrulline immunofluorescence were performed

To investigate the reaction of hemocytes and pericardial cells in the abdomens of *Cx. p. molestus, Cx. p. quinquefasciatus, Ae. albopictus*, and *Ae. aegypti* mosquitoes, individuals were infected with a median dose of 3.7 × 10^5^ CFU of live *E. coli* and controls were left untreated (Fig. 1). After approx. 24 h, the abdomens of the mosquitoes were dissected exposing the dorsal vessel (DV) and ventral nerve cord (VNC). The location of NOS at the time of fixation was detected by NADPHd activity staining. The activity of NOS up to the time of fixation is indicated by labeling the NO production by-product L-citrulline by immunofluorescence in the hemocytes and pericardial cells.

### Patterns of dorsal and ventral hemocytes

**Fig. 2:**
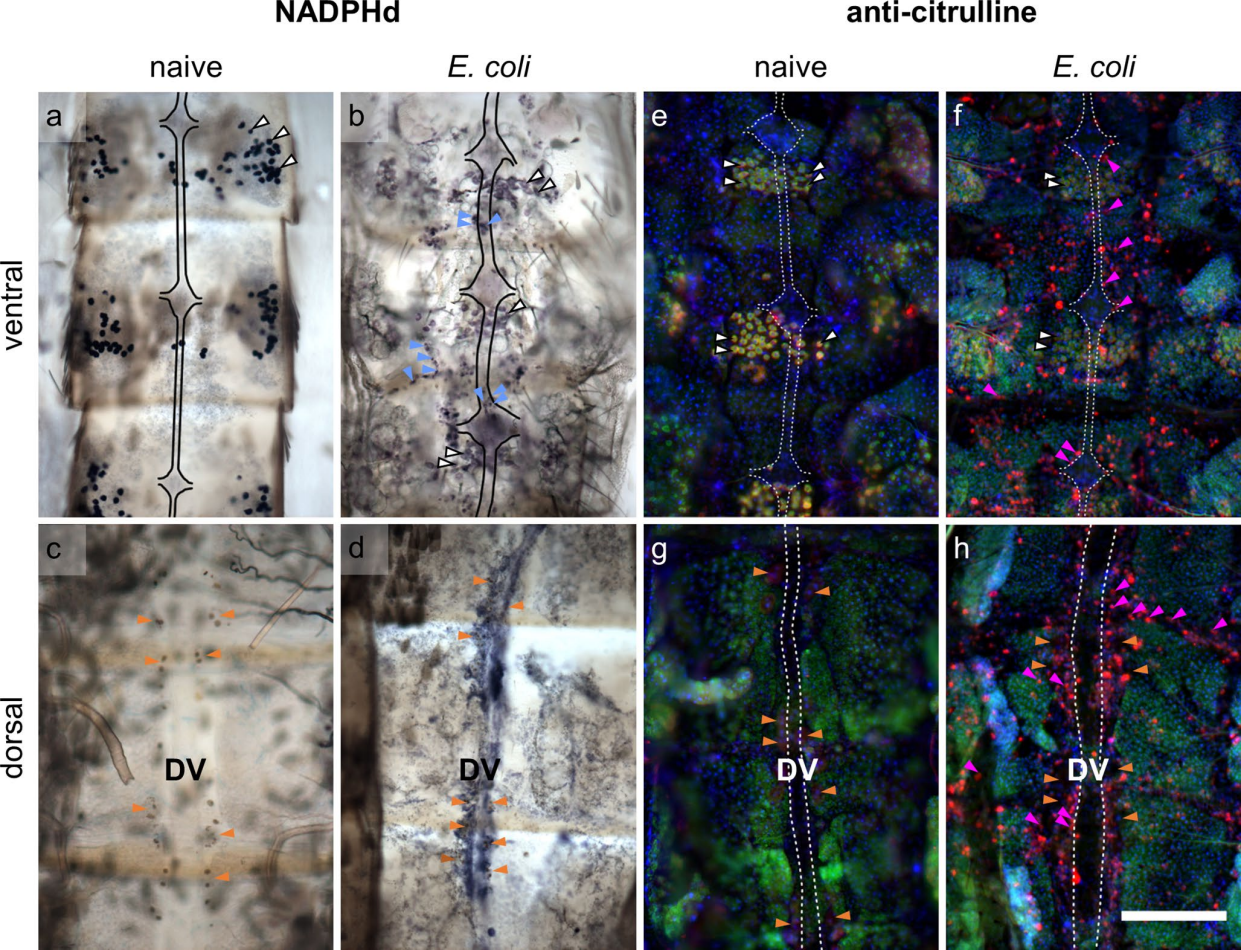
Representative dorsal and ventral abdomens of mosquitoes. **a**–**d** NADPHd activity and **e**–**h** anti-citrulline immunofluorescence of ventral abdomens (**a, b, e, f**) with the VNC indicated by a black or white dotted line and of dorsal abdomens (**c**, **d**, **g**, **h**) with the DV indicated by a white dotted line. **a**–**d** NADPHd activity shown by a blue staining of the oenocytes (white arrows) in ventral abdomens of naive (**a**, *Ae. albopictus*) and *E. coli*-infected mosquitoes (**b**, *Cx. p. molestus*) and in hemocytes (blue arrows) of infected mosquitoes ventrally (**b**) and especially around the DV (**d**, *Ae. albopictus*). Pericardial cells (orange arrows) near the DV are showing two dark spots (cytoplasmic inclusions (Leódido et al. 2013)) (**c**, *Cx. p. quinquefasciatus*). In the fluorescence images, pericardial cells are characterized by multiple nuclei (DAPI) (**g**, *Ae. albopictus*; **h**, *Cx. p. molestus*). **e**–**h** Anti-citrulline immunofluorescence (red) is showing in hemocytes (pink arrows) in ventral (**f**, *Cx. p. molestus*) and dorsal (**g**) abdomens of infected mosquitoes especially around the DV. Oenocytes are showing anti-citrulline and streptavidin (green) labeling (**e**, *Cx. p. molestus*). Anti-citrulline (red), streptavidin (green), DAPI (blue), scale bar: 250 µm

NADPHd-positive and citrulline-positive hemocytes were observed both dorsally and ventrally in the abdomens of the investigated mosquito species after bacterial infection (Fig. 2). In general, immune reactions were more pronounced in the dorsal part of the abdomens than on the ventral side, and naive mosquitoes rarely showed reactions (Table 1).

**Table 1:** Fraction of mosquito abdomens with NADPHd- or citrulline-positive hemocytes and melanization sites

		**NADPHd-positive hemocytes**				**Citrulline-positive hemocytes**				**Melanization**			
		**Ventral**		**Dorsal**		**Ventral**		**Dorsal**		**Ventral**		**Dorsal**	
		Fraction	*N*	Fraction	*N*	Fraction	*N*	Fraction	*N*	Fraction	*N*	Fraction	*N*
***Cx. mol***	Naive	0%	9	11%	9	0%	9	0%	9	22%	9	22%	9
	*E. coli*	20%	10	40%	10	63%	8	78%	9	89%	9	100%	9
***Cx. qui***	Naive	0%	12	0%	12	0%	12	0%	12	17%	12	33%	12
	*E. coli*	8%	13	31%	13	0%	12	0%	12	83%	12	100%	12
***Ae. alb***	Naive	0%	9	0%	9	0%	9	0%	9	11%	9	33%	9
	*E. coli*	10%	10	20%	10	11%	9	22%	9	33%	9	67%	9
***Ae. aeg***	Naive	0%	10	10%	10	0%	9	0%	9	0%	9	11%	9
	*E. coli*	20%	10	70%	10	44%	9	33%	9	89%	9	100%	9

Fractions (%) of individuals with positive hemocytes or melanization spots were calculated from total (*N*) (= 100%) of investigated individuals

In dorsal abdomens, hemocytes were mainly located near the DV (Fig. 2d, h). In preparations showing a strong immune response, hemocytes surrounded the entire DV, but also accumulated particularly at the segment boundaries and occasionally scattered throughout the entire dorsal half. Even in abdomens with weaker immune reactions, a pattern of hemocyte accumulation near the ostia of the DV was still observed (Online resource 1) (as previously shown by DiI labeling by Yan and Hillyer (2020) in *Anopheles*).

**Fig. 3:**
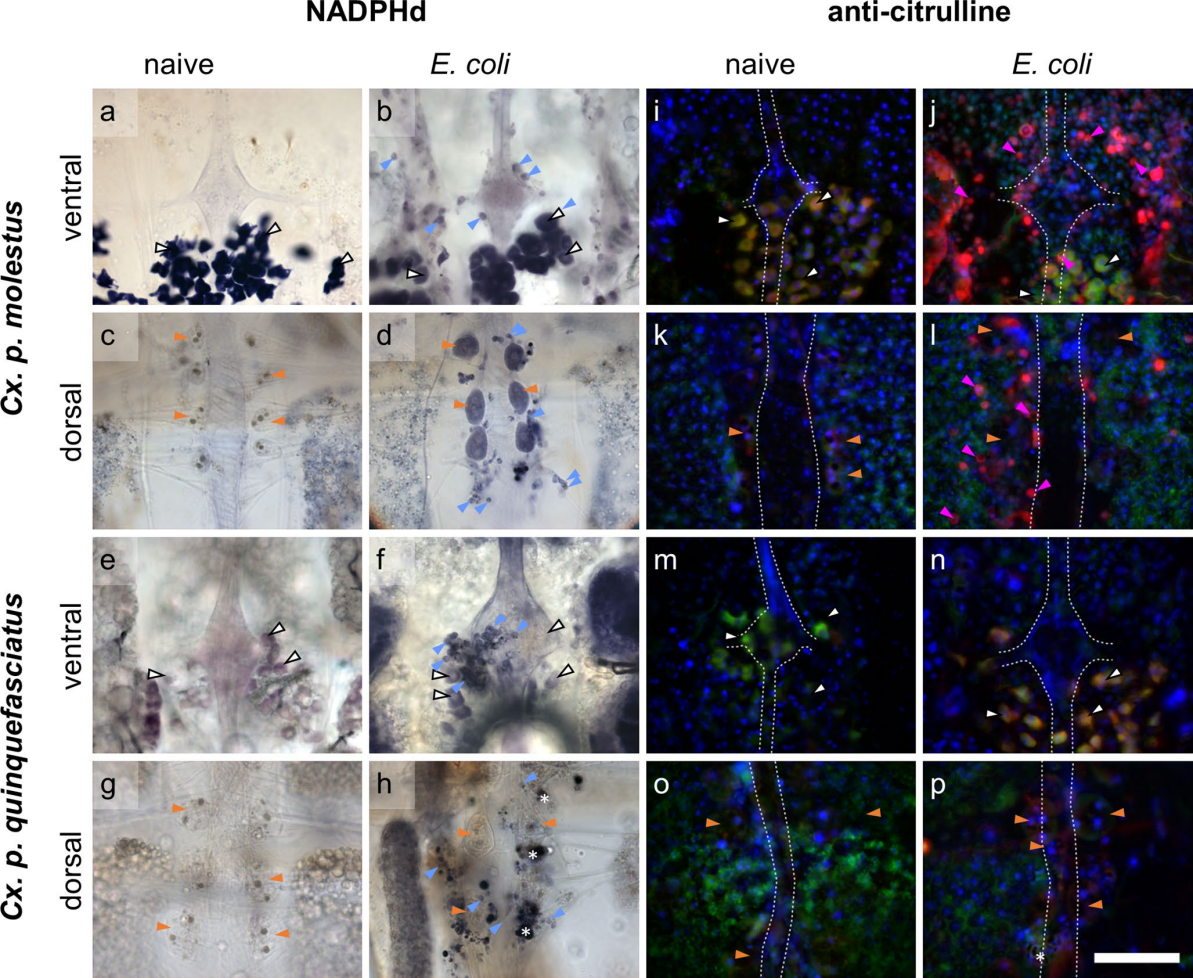
Details of dorsal and ventral abdomens of *Culex* mosquitoes. **a**–**h** NADPHd activity and anti-citrulline immunofluorescence (**i**–**p**) of *Cx. p. molestus* ventral (**a**, **b**, **i**, **j**) and dorsal (**c**, **d**, **k**, **l**) abdomens and *Cx. p. quinquefasciatus* ventral (**e**, **f**, **m**, **n**) and dorsal (**g**, **h**, **o**, **p**) abdomens. **a**–**h** NADPHd activity is showing in a blueish staining of the oenocytes (white arrows) in ventral abdomens of naive (**a**, **e**) and *E. coli*-infected mosquitoes (**b**, **f**) and in hemocytes (blue arrows) of infected mosquitoes near to the VNC (**b**, **f**) and around the DV (**d**, **h**). Pericardial cells (orange arrows) near the DV are showing two dark spots (cytoplasmic inclusions (Leódido et al. 2013)) (**c**, **d**, **g**, **h**) and a blue staining in *Cx. p. molestus* (**d**). Melanization is indicated by dark spots (white asterisks) (**h**). **i**–**p** Anti-citrulline immunofluorescence (red) is showing in hemocytes (pink arrows) in ventral (**j**) and dorsal (**l**) abdomens of infected mosquitoes. Pericardial cells (orange arrows) are characterized by multiple nuclei (DAPI). Oenocytes (white arrows) are showing anti-citrulline and strong streptavidin (green) labeling (**i**, **m**). VNC and DV are indicated by a dotted line. Anti-citrulline (red), streptavidin (green), DAPI (blue), scale bar: 100 µm

**Fig. 4:**
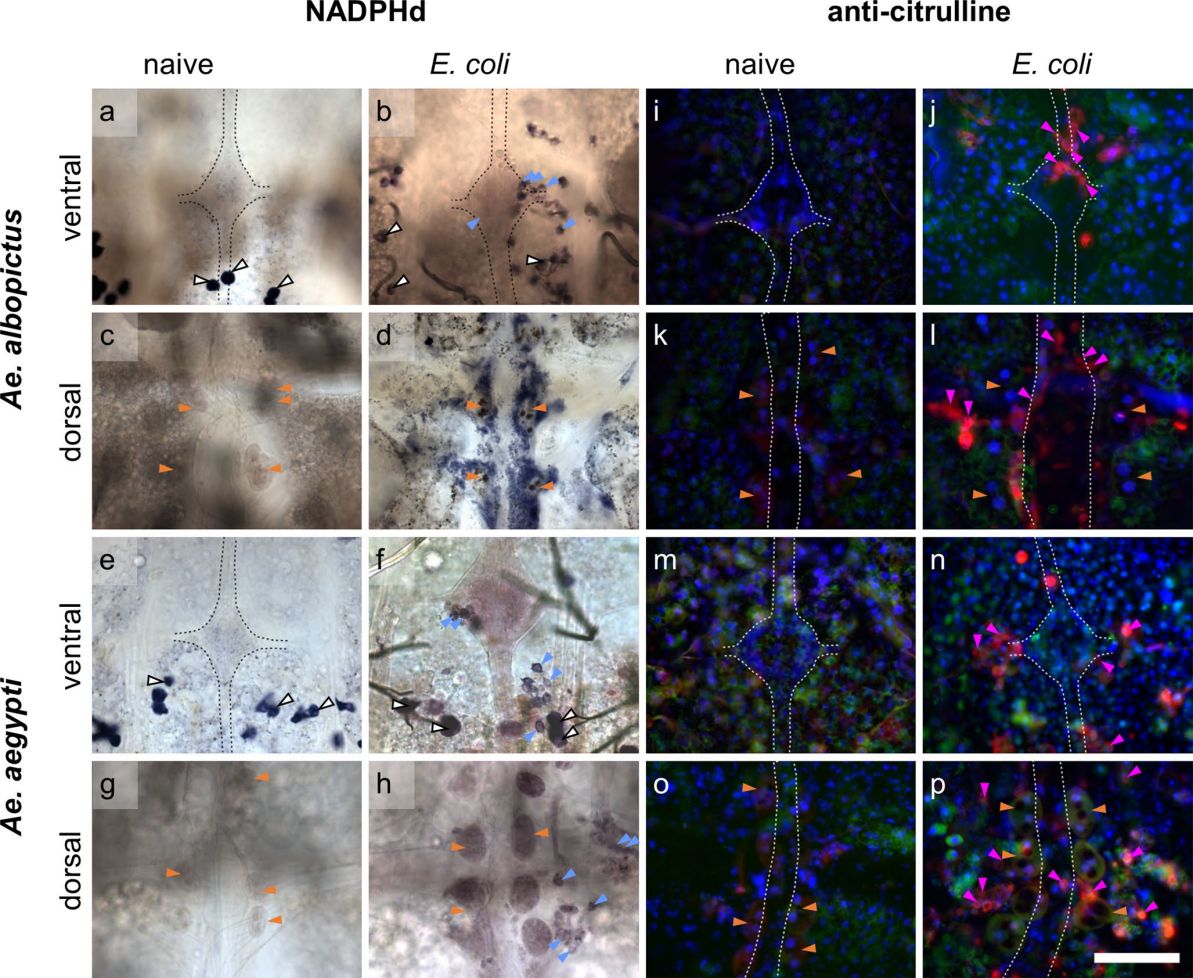
Details of dorsal and ventral abdomens of *Aedes* mosquitoes. **a**–**h** NADPHd activity and anti-citrulline immunofluorescence (**i**–**p**) of *Ae. albopictus* ventral (**a**, **b**, **i**, **j**) and dorsal (**c**, **d**, **k**, **l**) abdomens and *Ae. aegypti* ventral (**e**, **f**, **m**, **n**) and dorsal (**g**, **h**, **o**, **p**) abdomens. **a**–**h** NADPHd activity is showing in a blueish staining of the oenocytes (white arrows) in ventral abdomens of naive (**a**, **e**) and *E. coli*-infected mosquitoes (**b**, **f**) and in hemocytes (blue arrows) of infected mosquitoes near to the VNC (**b**, **f**) and around the DV (**d**, **h**). Pericardial cells (orange arrows) near the DV are showing two dark spots (cytoplasmic inclusions (Leódido et al. 2013)) (**c**, **d**, **g**, **h**) and a blueish staining in *Aedes aegypti* (**h**). VNC is indicated by a black dotted line. **i**–**p** Anti-citrulline immunofluorescence (red) is showing in hemocytes (pink arrows) in ventral (**j**, **n**) and dorsal (**l**, **p**) abdomens of infected mosquitoes. Pericardial cells show anti-citrulline labeling in *Aedes aegypti* and are characterized by multiple nuclei (DAPI). VNC and DV are indicated by a white dotted line. Anti-citrulline (red), streptavidin (green), DAPI (blue), scale bar: 100 µm

On the ventral sides of the abdomens, NADPHd-positive and citrulline-positive hemocytes were distributed across the entire abdominal half during a strong immune reaction (Fig. 2b, f). In addition, NADPHd- and citrulline-positive hemocytes were found close to or on the ganglia of the VNC (Figs. 3b, j, f and 4b, j, f, n).

### Comparison of immune responses in the different mosquito species

When comparing all four mosquito species, *Cx. p. molestus* and *Ae. aegypti* mosquitoes showed the highest rates of abdomens with NADPHd- and citrulline-positive hemocytes (Table 1). In contrast, in *Cx. p. quinquefasciatus*, no citrulline-positive hemocytes were observed (Table 1).

**Fig. 5:**
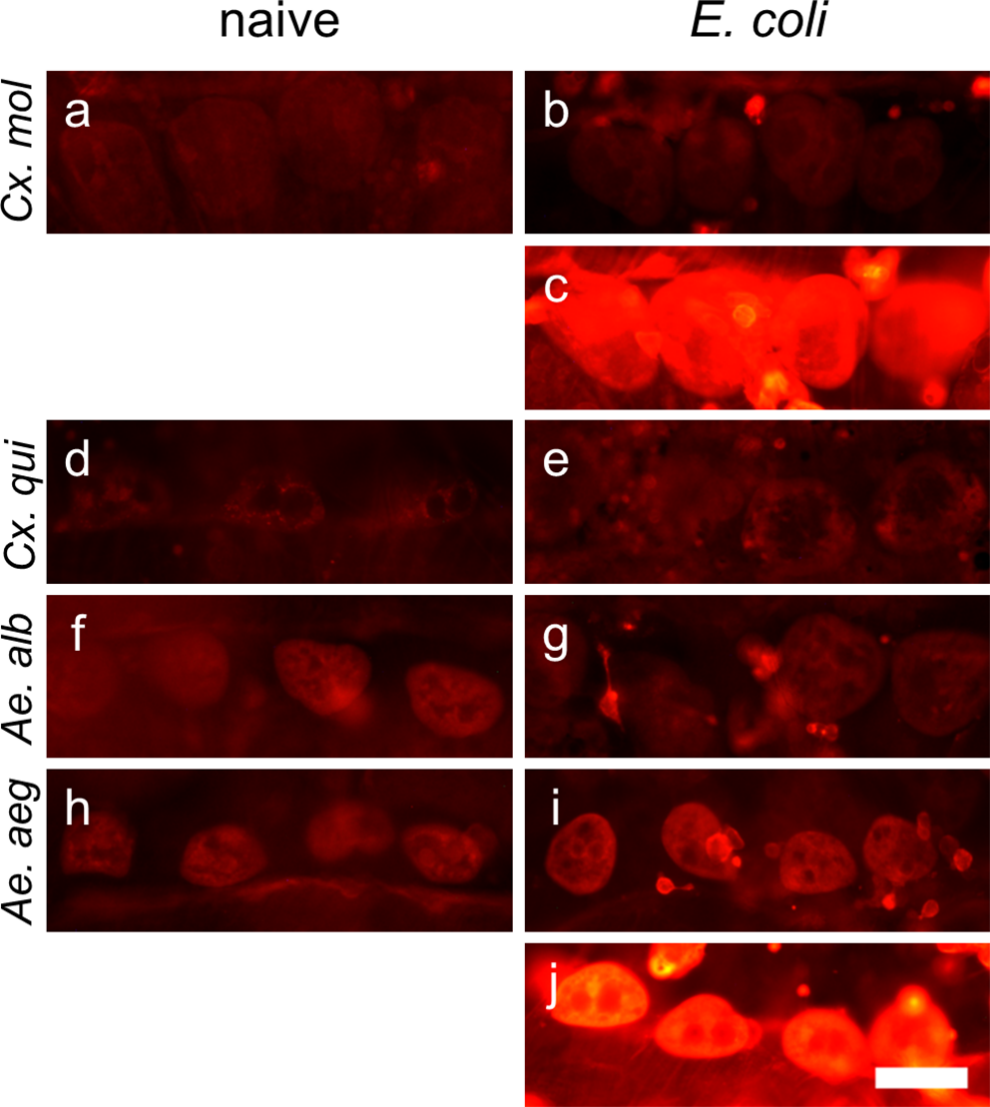
Comparison of anti-citrulline immunofluorescence intensity of pericardial cells in mosquitoes. Pericardial cells of all species investigated showed basic anti-citrulline immunofluorescence in naive mosquitoes (**a**, **d**, **f**, **h**) and for *Cx. p. quinquefasciatus* (**e**) and *Ae. albopictus* (**g**) in infected animals. Immunofluorescence intensity varies between the individuals within the species *Cx. p. molestus* (**b**, **c**) and *Ae. aegypti* (**i**, **j**). All images taken with identical settings at 1000-ms exposure time. Scale bar: 50 µm

Pericardial cells surrounding the DV were distinguishable from other cells by their location next to the DV and their size as well as characterized by multiple nuclei in immunofluorescence and two brown cytoplasmic inclusions in light microscopy (as previously reported for *Ae. aegypti* by Leódido et al. (2013)). Usually, pericardial cells showed no diaphorase activity (Figs. 2c, d, 3c, g, h and 4c, d, g). In some preparations, a blue staining was observed (Figs. 3d and 4h, Online resource 1c), but no clear connection between the staining intensity and mosquito species or infection status was found (data not shown). With anti-citrulline labeling, a certain background fluorescence of the pericardial cells was revealed in all species, whether treated or not (Fig. 5). Notably, *Cx. p. molestus* and *Ae. aegypti* mosquitoes frequently exhibited more varying and higher fluorescence intensity levels in infected individuals (Fig. 5b, c, i, j). These infected individuals can present pericardial cells showing elevated immunofluorescence intensity (Fig. 5c, j), and other individuals express rather background levels of L-citrulline immunofluorescence (Fig. 5b, i). *Cx. p. molestus* mosquitoes have a 3.78 times wider range of intensity values than naive individuals, and 43% of intensity values of infected individuals were above the 75% percentile of naive values (Online resource 2). Similarly, infected *Ae. aegypti* showed a 2.57 times wider range of intensity values, and 57% of intensity values were above the 75% percentile of naive (Online resource 2).

**Fig. 6:**
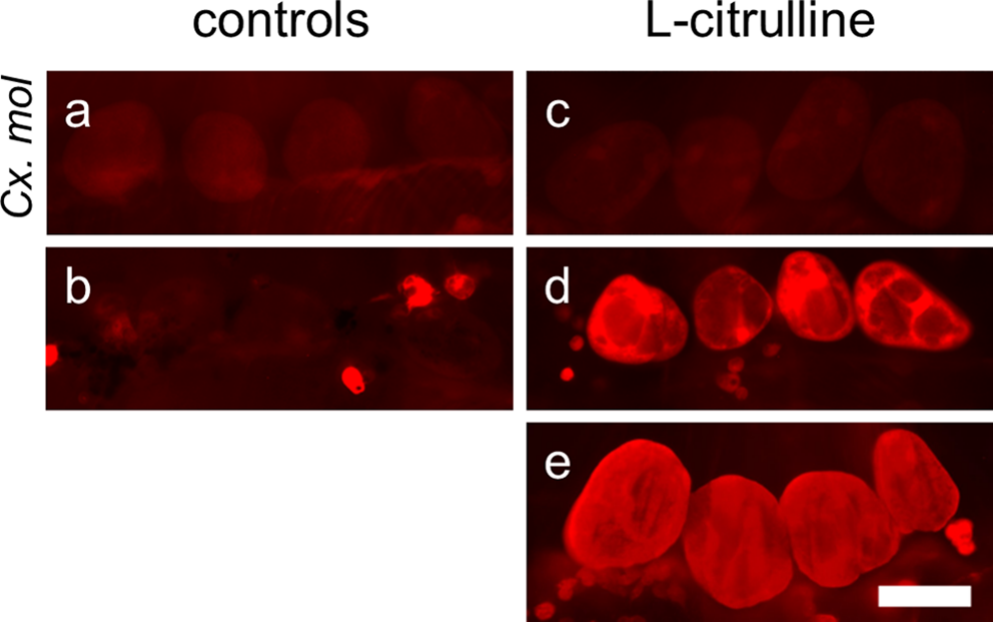
Incubation of *Cx p. molestus* pericardial cells with the amino acid L-citrulline. Higher concentrations of L-citrulline in Ringer solution (**d** 5 µM, *n* = 3; **e** 50 µM, *n* = 3) produced increased anti-citrulline immunofluorescence intensity in pericardial cells compared to lower concentrations (**c** 500 nM, *n* = 3). The fluorescence intensity of the lowest concentration (**c**) matched the intensity of the controls (**a** naive, *n* = 3; **b** inactivated *E. coli*, *n* = 10). All images were taken with identical settings at 1000-ms exposure time. Scale bar: 50 µm

Since pericardial cells are known for their ability to filtrate the hemolymph, we tested if the amino acid L-citrulline produced by the hemocytes and potentially secreted into the hemolymph contributes to the occasional high anti-citrulline immunofluorescence intensity in pericardial cells. Abdomens of *Cx. p. molestus* mosquitoes were dissected and incubated with different concentrations of L-citrulline in Ringer solution (Fig. 6c (500 nM), d (5 µM), e (50 µM)). Compared to controls (Fig. 6a (naive), b (*E. coli* inactivated)), preparations with 500 nM L-citrulline exhibited a similar anti-citrulline immunofluorescence intensity. With increasing L-citrulline concentrations, the immunofluorescence intensity was also increasing. Notably, at 50 µM L-citrulline, swelling of the pericardial cells can be observed showing elongated folds mirroring the directions of the muscle fibers from lateral to the periosteal region (Fig. 6e). Additionally, the hemocytes become visible at high L-citrulline concentrations (Fig. 6d, e). The anti-citrulline antibody was tested for sensitivity and can detect L-citrulline concentrations down to 10 µM in dot blot immune assay (Online resource 3).

The melanization reaction, shown in black capsules (Fig. 3h) or brown aggregations (Online resource 4 and 5), was found in all tested species with high fractions between 67 and 100% in dorsal abdomens and lower fractions between 33 and 89% in ventral abdomens in infected mosquitoes (Table 1). Whereas *Ae. albopictus* was not only exhibiting the lowest melanization reaction in *E. coli*-infected individuals but also relatively low fractions of abdomens with NADPHd- and citrulline-positive hemocytes (Fig. 1), they show a pronounced fat body and a slight tendency towards bluish or brownish staining of the fat body when infected (Fig. 2d, Online resource 1c).

Furthermore, on the ventral side, a type of cells was identified as oenocytes due to their location and regular appearance pattern on the fat body. Oenocytes were mostly found beneath the ganglia in each abdominal segment, extended across the entire width of the fat body and reaching the pleural membrane (Fig. 2a, e, Online resource 6). They exhibited a strong NADPHd staining (e.g., Fig. 3a) and anti-citrulline immunofluorescence (e.g., Fig. 3n, Online resource 6b, d, f, h, j, l, n, p) as well as streptavidin fluorescence (e.g., Fig. 3n, Online resource 6a, c, e, g, l, k, m, o). Some fluorescence in the red channel was already showing when only applying the secondary antibody (Online resource 7). However, no changes in the intensity due to infection were observed in any of the mosquito species.

## Discussion

In this study, we investigated the immune response in four important vector species, *Aedes aegypti*, *Aedes albopictus*, *Culex p. molestus*, and *Culex p. quinquefasciatus*, focusing on the expression of NOS and the production of NO in immune-relevant cell types following bacterial challenge and potential effects on the central nervous system. Our research revealed both similarities and differences in the immune response across mosquito species.

### Common patterns of NOS-positive and NO-producing hemocytes in the abdomens

We observed an increased expression of NOS in mosquito hemocytes, as demonstrated by NADPHd staining in all examined mosquito species. Notably, NADPHd-positive hemocytes were particularly concentrated near the heart, exhibiting a pattern similar to that reported for bacterial-infected *Anopheles* (Estévez-Lao et al. 2020). These hemocytes were found in the vicinity of ostia and pericardial cells, therefore termed periostial hemocytes (King and Hillyer 2012). Accumulation of hemocytes due to the high hemolymph flow in these areas visualized by cell-labeling dye DiI has been previously reported in *Aedes aegypti*, *Aedes albopictus*, and *Culex* spp. (Yan and Hillyer 2020). To the best of our knowledge, we are the first to demonstrate NADPHd-positive periostial hemocytes in the investigated *Aedes* and the *Culex* species. A corresponding pattern was shown by citrulline immunofluorescence, providing evidence of NOS activity in three out of four examined mosquito species. Hemocytes positive for NADPHd and citrulline were widely distributed along the abdominal walls during pronounced immune reactions, as previously shown for *Locusta migratoria* (Bergmann et al. 2021). In mosquitoes, these hemocytes tend to accumulate between the fat body lobes, where both circulating bacteria and hemocytes may be less effectively reached by the hemolymph flow. Some aspects of the immune response in mosquitoes could be associated with injury. In transcriptomic analysis, sterile injury in *Anopheles* mosquitoes produced expression changes shared with septic injury (Dimopoulos et al. 2002). However, a distinctive gene cluster, indicative of the response to bacterial infection, was activated only in the septic treatment. When analyzing locust abdomens, scarce instances of NO-producing hemocytes after sham injection were observed (Bergmann et al. 2021). We consider the impact of a small injection in the thorax to be a minor factor compared to the introduction of live bacteria distributed throughout the body cavity, influencing the hemocyte response in the abdomen.

Furthermore, hemocytes were not only present on the dorsal side of the abdomen but also on the ventral side near the VNC. While previous studies mostly focused on the dorsal region as the site of hemocyte immune response (King and Hillyer 2012; Yan and Hillyer 2020), our study, in line with previous findings in locusts (Bergmann et al. 2021), demonstrates a considerable portion of the immune response occurring on the ventral side. Although NADPHd- and citrulline-positive hemocytes as well as melanized inclusions were less frequently observed ventrally than dorsally, labeled hemocytes were often located directly near the VNC in infected mosquitoes. Since NO is an important neurotransmitter (Bicker 1998), the proximity of NO-producing hemocytes to the central nervous system raises questions about potential immunomodulatory effects. In grasshoppers, stimulated and NO-producing hemocytes trigger the accumulation of cGMP in neurons of the VNC within the canonical NO/cGMP pathway (Bergmann et al. 2021).

### Differences between the mosquito species

Although an infection with *E. coli* in the tested mosquitoes resulted in a higher proportion of abdomens with NADPHd- and citrulline-positive hemocytes and an increase in melanization, among the examined parameters, *Culex p. molestus* and *Aedes aegypti* exhibited a pronounced immune response.

Pericardial cells, also known as nephrocytes, are distributed around the DV, taking up potential toxins from the hemolymph and thus encountering signaling molecules and immune peptides (De Das et al. 2018; Wigglesworth 1970). Indeed, pericardial cells and unstimulated hemocytes were able to take up the amino acid L-citrulline, when applied in extremely high concentrations (5 and 50 µM) indicating a passive mode of transportation. The swelling of the pericardial cells may be explained by an altered osmolarity of the cells compared to the extracellular fluid and thus water influx. In contrast, 500 nM L-citrulline—a still unphysiologically high concentration—was not able to increase the anti-citrulline immunofluorescence intensity above background level. The physiological range of NO in the extracellular fluid is estimated ranging between 100 pM and 5 nM and in the case of activated macrophages may increase to about 10 nM (Hall and Garthwaite 2009). In the cell culture of macrophages, L-citrulline concentrations between 10 and 12 nM were reached after 12 h (Kröncke et al. 1993). Upon comprehensive examination, it is improbable that the physiological concentrations of extracellular L-citrulline exert a significant influence on the fluorescence intensity of pericardial cells during infection.

Moreover, pericardial cells express various immune-related genes, including lysozyme c-1 and cecropin, in response to immune stimulation in mosquitoes (Cardoso-Jaime et al. 2021, 2023). In infected *Culex p. molestus* and *Aedes aegypti*, pericardial cells exhibit increased citrulline immunofluorescence compared to naive individuals. The specificity of the anti-citrulline antibody for the amino acid L-citrulline, as shown in pre-adsorption controls in locusts (Bergmann et al. 2021), supports the assumption of L-citrulline accumulation in pericardial cells and thus NO production. Furthermore, some individuals also demonstrated strong NADPHd activity, providing further indication of the involvement of pericardial cells in the immune response. However, unlike observations on infected *Anopheles* mosquitoes (Cardoso-Jaime et al. 2023), we did not observe swelling of the pericardial cells in infected mosquitoes.

In contrast to *Culex p. molestus* and *Aedes aegypti*, *Aedes albopictus* showed a comparatively weaker immune response, and *Culex p. quinquefasciatus* displayed no citrulline-positive hemocytes. Since pathogens, when small enough, are primarily phagocytosed, it is possible that *Aedes albopictus* and *Culex p. quinquefasciatus* efficiently intercept and eliminate *E. coli* from the hemolymph, superseding further immune response by hemocytes (Ratcliffe and Rowley 1979; Da Silva et al. 2000). Therefore, the lower proportion of melanized abdomens in *Aedes albopictus* may suggest that melanization becomes necessary when hemocyte phagocytic capacity is depleted. Moreover, *Aedes albopictus* individuals exhibited a well-developed fat body, occasionally NADPHd positive, which may contribute to pathogen defense through the release of antimicrobial peptides (De Das et al. 2018, Herrera-Ortiz et al. 2011; Hillyer and Estévez-Lao 2010). It is yet to be determined if this represents species-specific traits or arises from distinct reactions to identical rearing conditions.

In comparison to infected mosquitoes, it is noteworthy that a certain percentage of melanization was observed even in naive mosquitoes. This can be attributed to the fact that mosquitoes were not kept under sterile conditions, and the water reservoirs of larvae and the food sources of adult mosquitoes were likely inhabited by various microorganisms under warm and humid rearing conditions. This may lead to an elevated immune reaction in some individuals who were primed by a naturally acquired infection (Moreno-García et al. 2015). As mosquito cell culture experiments have demonstrated, immune priming can also occur as a result of viral infection (Laureti et al. 2023). In addition, naive *Culex p. molestus* and *Aedes aegypti*, albeit at low proportion, showed abdomens with NADPHd-positive hemocytes.

The expression of immune genes is subject to strong and environment-dependent selection pressure (Larragy et al. 2023). Although some components of the immune system, such as the Toll pathway, exhibit remarkable conservation across insects and even vertebrates (Rock et al. 1998), the specific expression of immune pathways can vary substantially among different insect species. This variation is exemplified by the pea aphid, which possesses a reduced immune system lacking the IMD pathway (International Aphid Genomics Consortium 2010). However, differences were also observed among mosquito species. *Aedes albopictus*, for instance, has the largest genome among sequenced mosquitoes, attributed, among other factors, to an expansion in the number of immune genes (Chen et al. 2015). Similarly, *Culex p. quinquefasciatus* displays an expansion in the number of immune genes compared to *Anopheles gambiae* and *Aedes aegypti*, particularly in gene families like C-type lectins, fibrinogen-related proteins, and serine protease inhibitors (Bartholomay et al. 2010). Serine protease inhibitors play critical roles in immune pathway regulation (Shakeel et al. 2019) by influencing various immune components, including the downregulation of antimicrobial peptides (Han et al. 2014) and the Toll and prophenoloxidase pathways (Li et al. 2016). Moreover, differences in immune responses to fungal infections were observed in *Aedes albopictus* and *Culex pipiens* mosquitoes, affecting the direction and intensity of immune pathway component expression (Ramirez et al. 2021). Similarly, *Culex p. quinquefasciatus* produces fewer virus-derived small interfering RNAs compared to *Aedes aegypti* when infected with Rift Valley fever virus (Dietrich et al. 2017).

### Oenocytes of all species show strong labeling

In contrast, oenocytes consistently exhibit strong NADPHd staining and citrulline immunofluorescence. Similar to findings in *Aedes albopictus*, *Aedes aegypti*, and *Culex p. quinquefasciatus*, *Culex p. molestus’* oenocytes are predominantly located at the periphery of the fat body (Martins et al. 2011a). Oenocytes are characterized by a high protein content, strong synthesis activity, and a homogeneous cytoplasm mainly filled by smooth endoplasmic reticulum (ER) (Martins et al. 2011a; Martinson et al. 2022). Additionally, oenocytes play a role in hemocyte differentiation and maintaining elevated immune responses in primed mosquitoes (Gomes et al. 2021). Besides expressing various immune-related genes, mosquito oenocytes also express NADPH cytochrome P450 reductase, an ER protein (Martins et al. 2011b). The enzyme is also displaying diaphorase activity after fixation (Young et al. 1997). It must be pointed out that naive individuals show oenocytes with a strong NADPHd activity and citrulline immunofluorescence as well, suggesting that it is more likely attributed to elevated protein synthesis and enzyme activity rather than being linked to an infection. However, the fluorescence in the red channel was already showing to some extent, when only applying the secondary antibody. Thus, the oenocytes may be prone to unspecific labeling.

## Conclusion

**Fig. 7:**
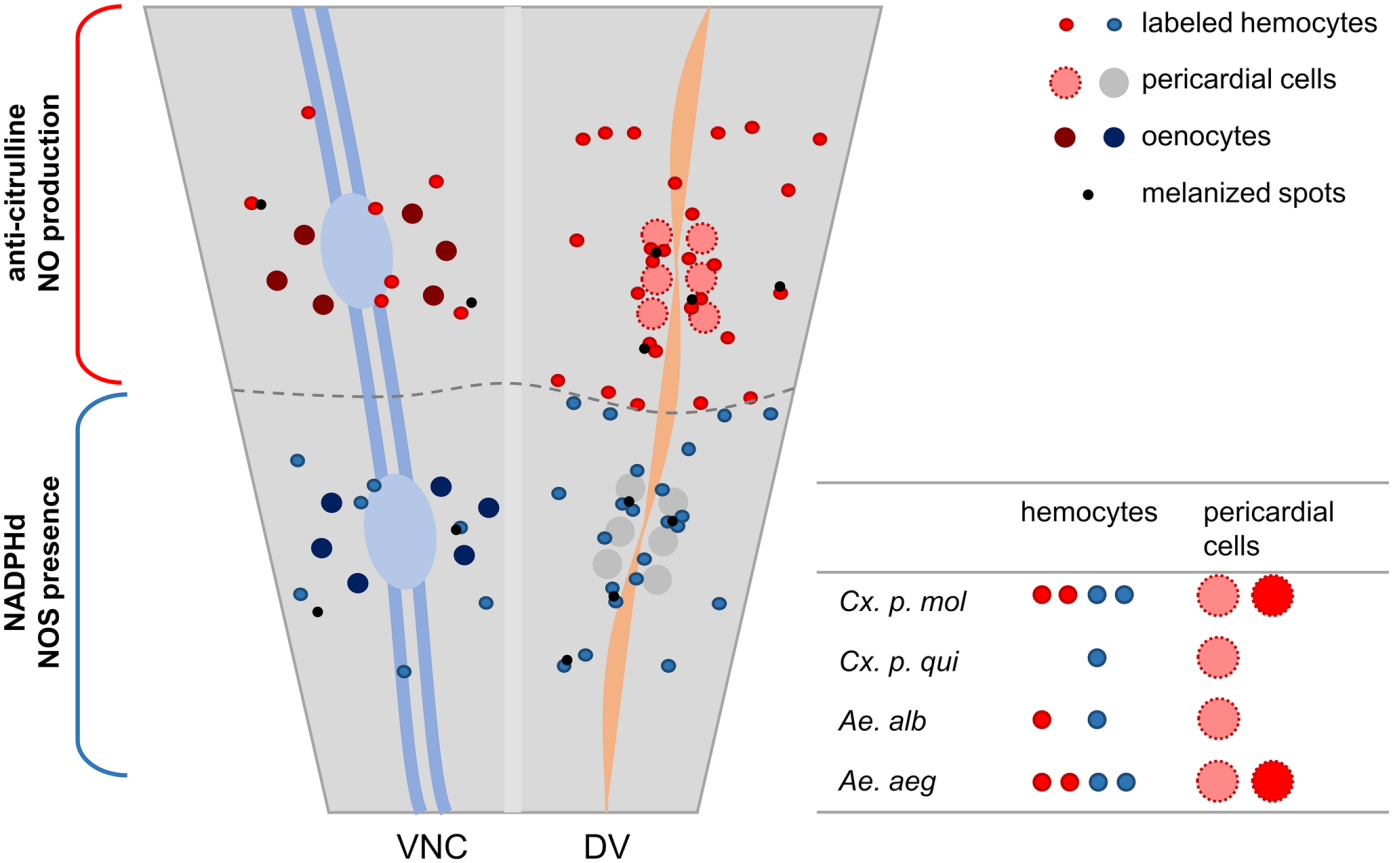
Schematic overview of hemocyte distribution patterns of *E. coli*-infected mosquitoes. NADPHd-stained hemocytes (blue-encircled cells) are indicative of NOS presence, while anti-citrulline immunofluorescence indicates NO production in labeled hemocytes (red-encircled cells). Labeled hemocytes are present on dorsal abdomens, around the DV as well as on the ventral side near or directly on the VNC and often co-occur with melanized spots (black dots). Fractions of abdomens with positive hemocytes were highest in *Cx. p. molestus* and *Ae. aegypti* mosquitoes. No NO-producing hemocytes were observed in *Cx. p. quinquefasciatus*. Pericardial cells (red circles with dotted lines) exhibit varying anti-citrulline intensity in infected *Cx. p. molestus* and *Ae. aegypti* mosquitoes. Oenocytes (dark red or blue filled circles) display strong labeling independent of infection status

In conclusion, the parallel comparison of these four vector species provides valuable insights into their hemocytes’ reaction during *E. coli* infection. These findings align with previous research on location and NOS expression in *Anopheles* hemocytes (King and Hillyer 2012, 2013; Estévez-Lao et al. 2020), suggesting a widespread phenomenon across diverse species, including grasshoppers (Bergmann et al. 2021; Yan and Hillyer 2020) even on ventral abdomens and the VNC (Fig. 7). Nevertheless, we did find differences between immune reactions between mosquito species and even larger differences between members of the same genus than between genera (Fig. 7). Considering the dynamic shifts in vector species composition and pathogen distribution anticipated, the need for further species-specific investigation of vector immunity and possible interactions of pathogens and the nervous system becomes even more urgent. This would contribute to a better understanding of vector competence and targeted alternative (non-insecticide) strategies for vector control and disease prevention.

### Supplementary Information

Below is the link to the electronic supplementary material.

Supplementary file1 (TIF 3621 KB)

Supplementary file2 (XLSX 25 KB)

Supplementary file3 (TIF 2489 KB)

Supplementary file4 (TIF 4409 KB)

Supplementary file5 (TIF 4701 KB)

Supplementary file6 (TIF 3318 KB)

Supplementary file7 (TIF 3354 KB)

## Data Availability

The data supporting our findings are available from the corresponding author upon reasonable request.
