# Supplementary figures and images for: Localization of nitric oxide–producing hemocytes in Aedes and Culex mosquitoes infected with bacteria
Source: Cell Tissue Res. 2024 Jan 19;395(3):313–26. doi: 10.1007/s00441-024-03862-1 (PMC10904431; doi:10.1007/s00441-024-03862-1)

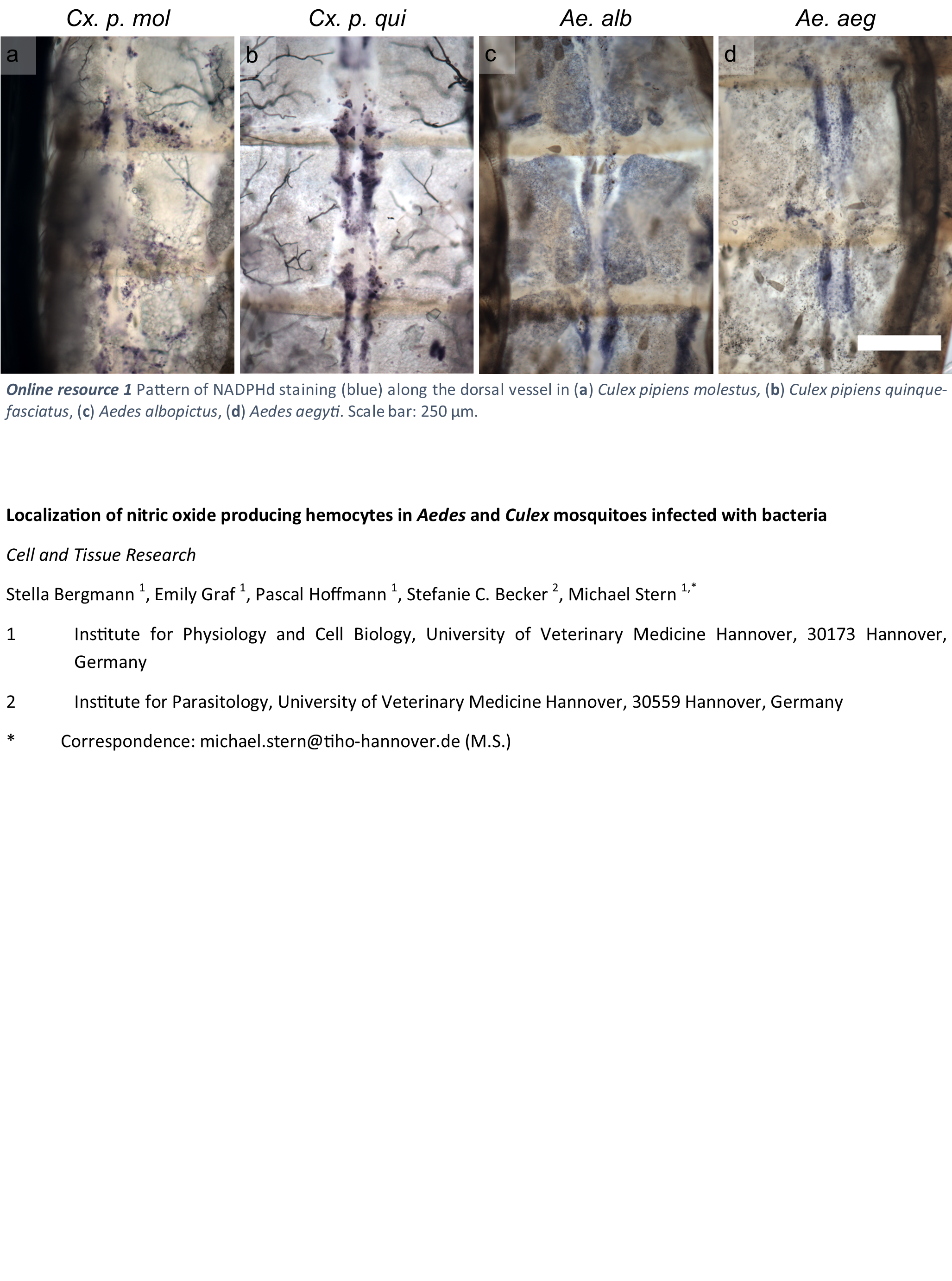

Supplement: Supplementary file 1 — Supplementary file1 (TIF 3621 KB) [file 441_2024_3862_MOESM1_ESM.tif]

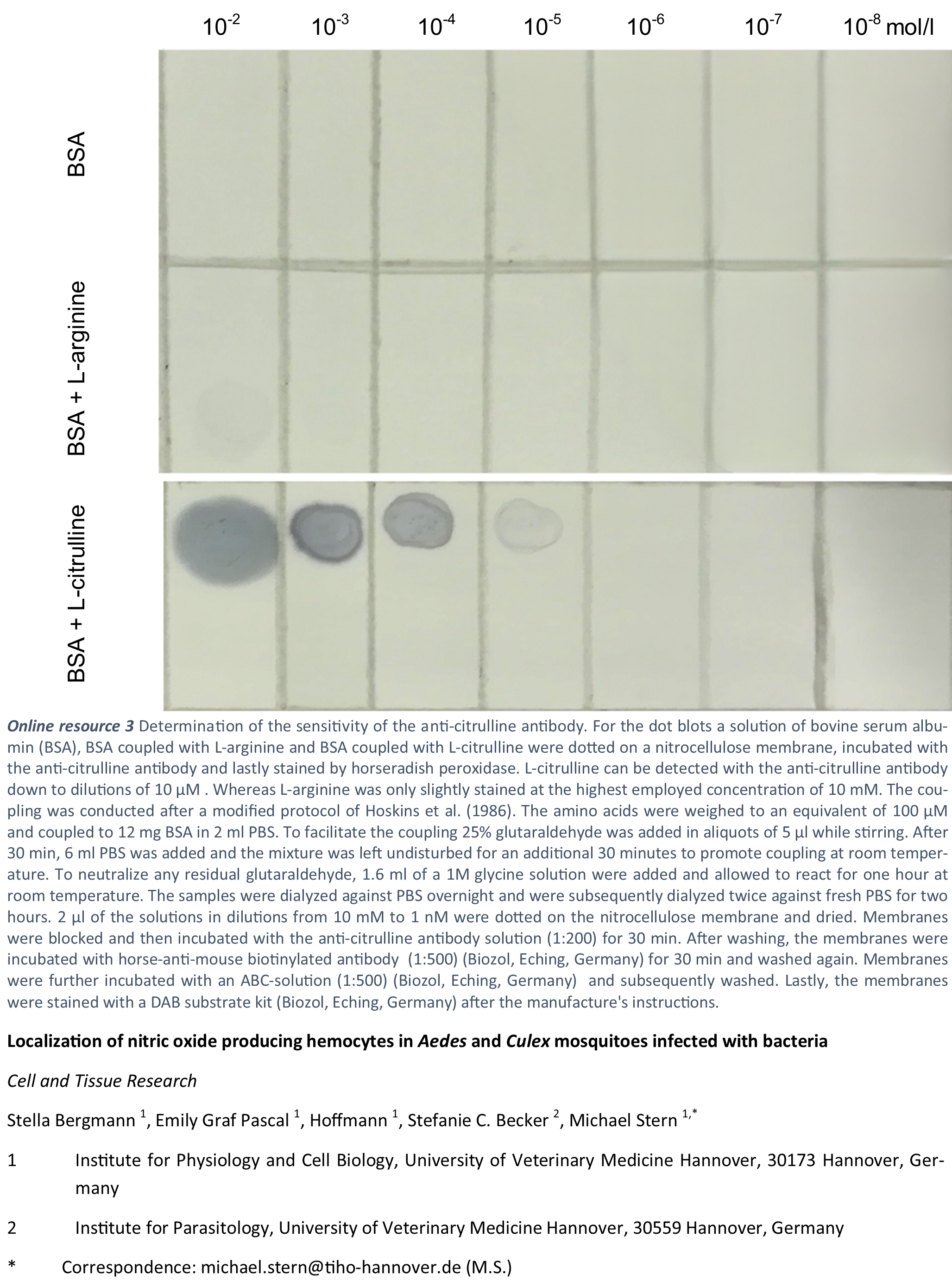

Supplement: Supplementary file 3 — Supplementary file3 (TIF 2489 KB) [file 441_2024_3862_MOESM3_ESM.tif]

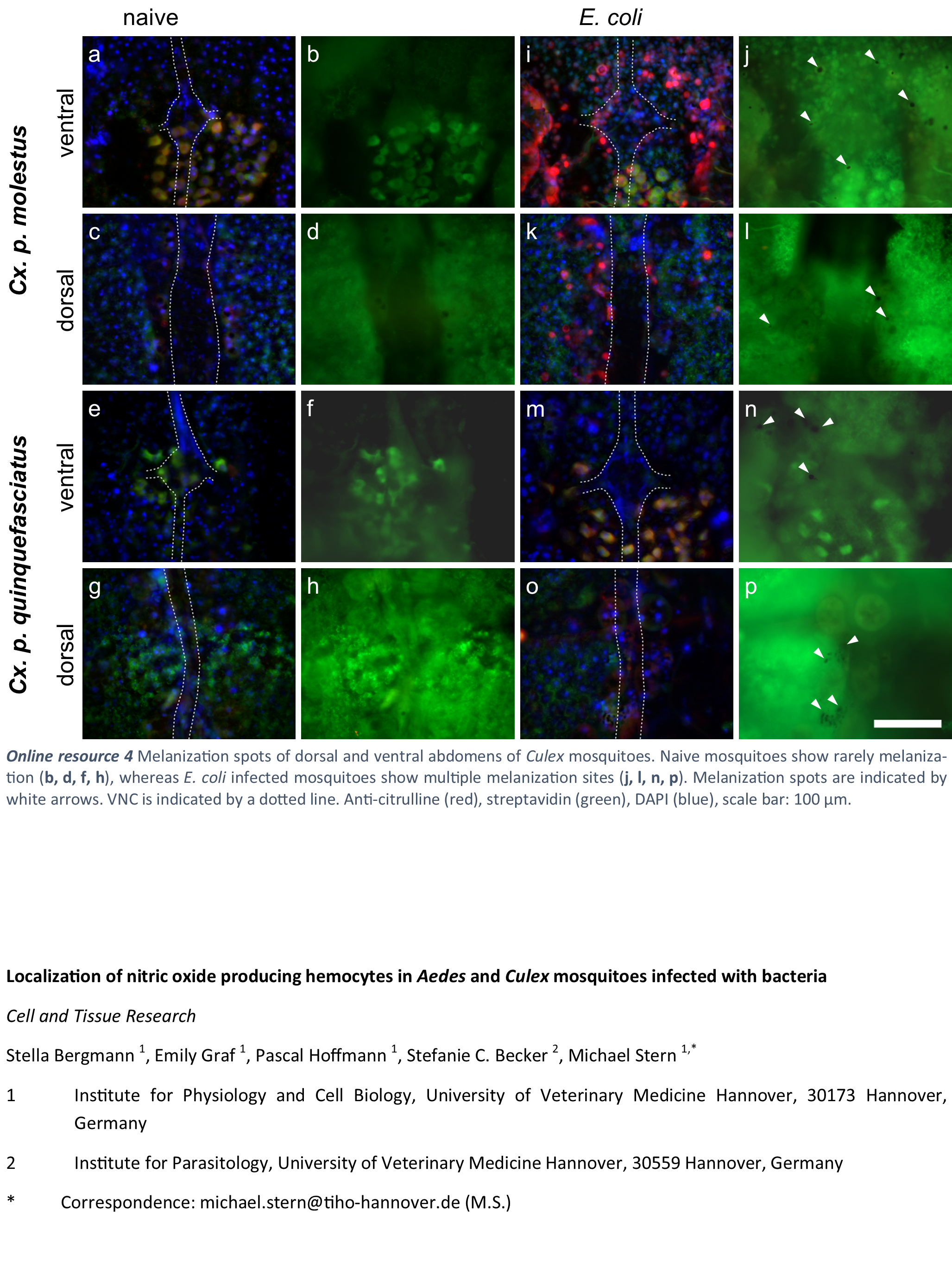

Supplement: Supplementary file 4 — Supplementary file4 (TIF 4409 KB) [file 441_2024_3862_MOESM4_ESM.tif]

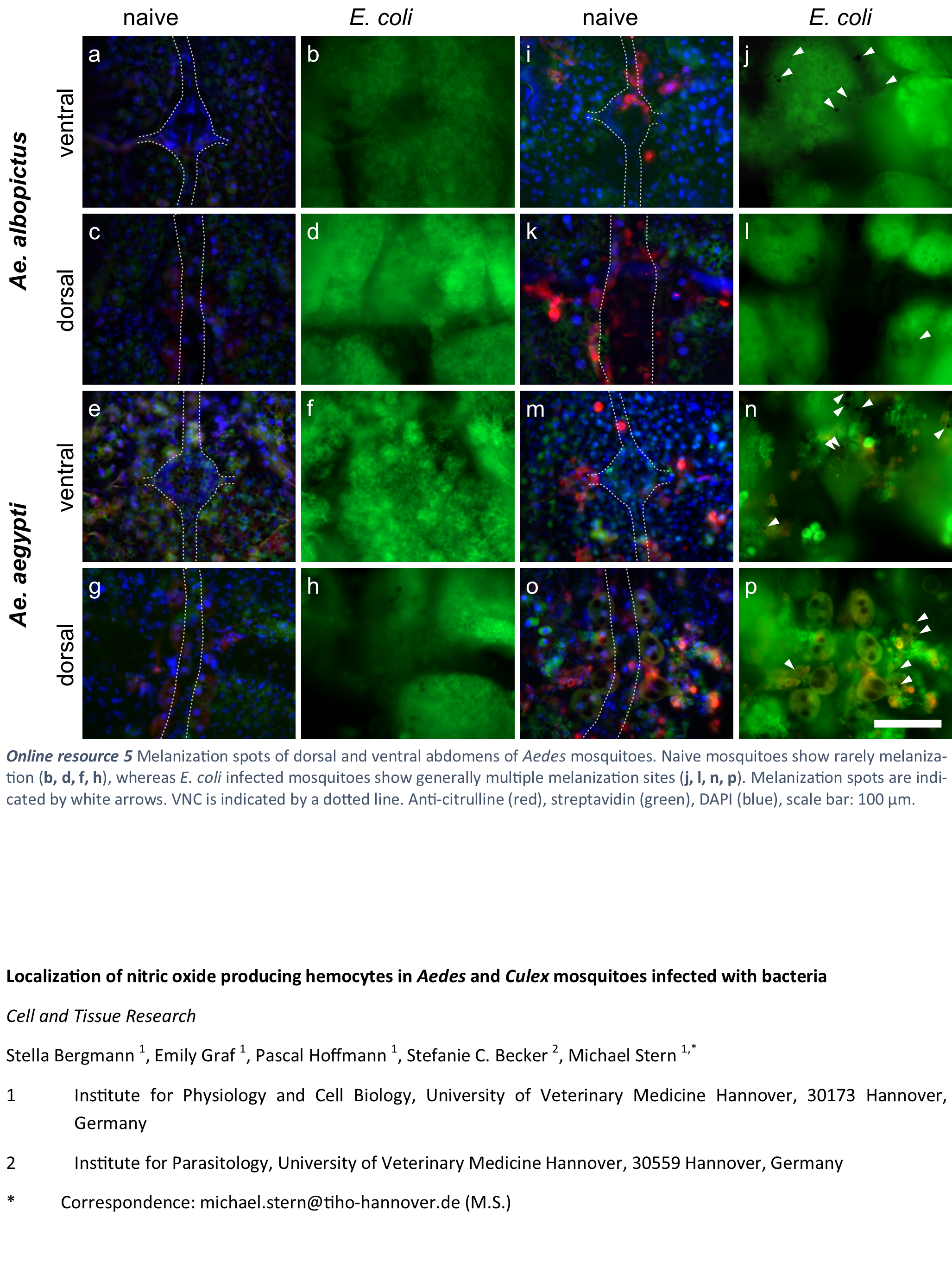

Supplement: Supplementary file 5 — Supplementary file5 (TIF 4701 KB) [file 441_2024_3862_MOESM5_ESM.tif]

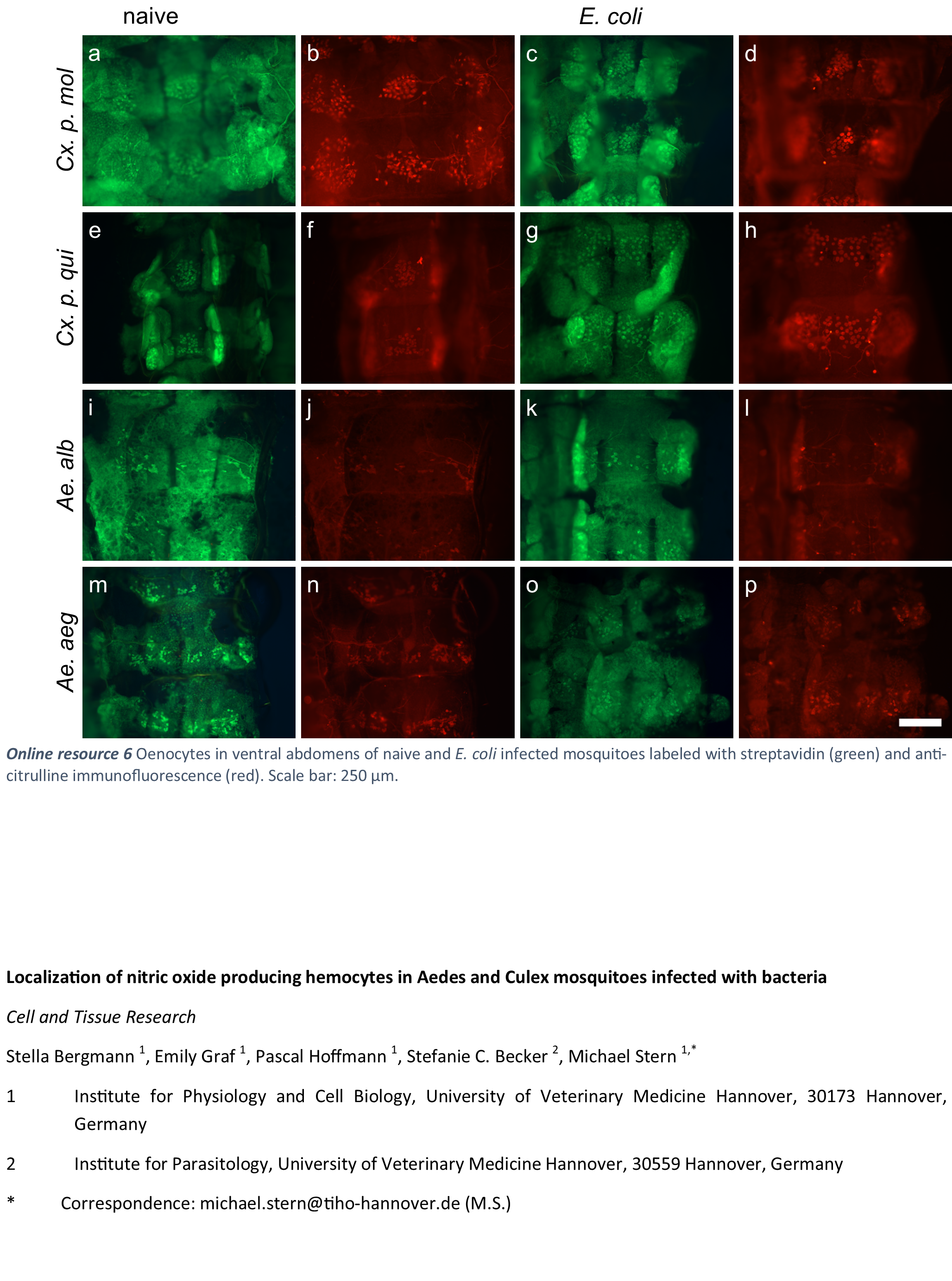

Supplement: Supplementary file 6 — Supplementary file6 (TIF 3318 KB) [file 441_2024_3862_MOESM6_ESM.tif]

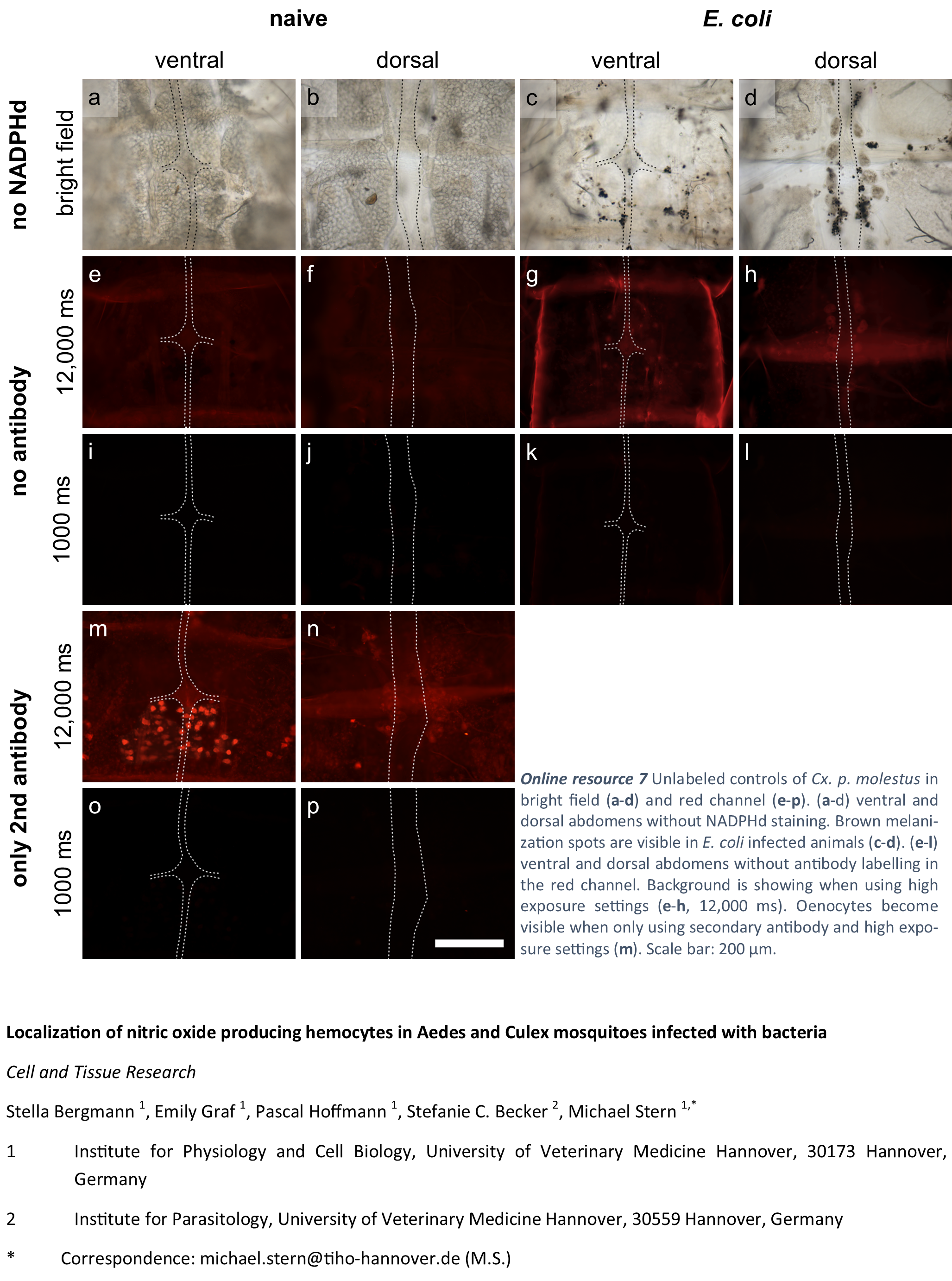

Supplement: Supplementary file 7 — Supplementary file7 (TIF 3354 KB) [file 441_2024_3862_MOESM7_ESM.tif]
